# Scaling-dependent tunability of spin-driven photocurrents in magnetic metamaterials

**DOI:** 10.1515/nanoph-2025-0514

**Published:** 2025-12-15

**Authors:** Gabriele Cavanna, Hidehisa Taketani, Hikaru Watanabe, Da Pan, Anna Honda, Daiki Oshima, Takeshi Kato, Masakazu Matsubara

**Affiliations:** Division of Materials Physics, Graduate School of Engineering Science, The University of Osaka, Toyonaka, Osaka 560-8531, Japan; Department of Physics, Graduate School of Science, Tohoku University, Sendai 980-8578, Japan; Department of Physics, The University of Tokyo, Hongo, Bunkyo-ku, Tokyo 113-0033, Japan; Institute of Materials and Systems for Sustainability, Nagoya University, Furo-cho, Chikusa-ku, Nagoya 464-8603, Japan; Department of Electronics, Nagoya University, Furo-cho, Chikusa-ku, Nagoya 464-8603, Japan; Center for Spintronics Research Network (CSRN), Graduate School of Engineering Science, The University of Osaka, Toyonaka, Osaka 560-8531, Japan; Spintronics Research Network Division, Institute for Open and Transdisciplinary Research Initiatives, The University of Osaka, Suita, Osaka 565-0871, Japan; PRESTO, Japan Science and Technology Agency (JST), Kawaguchi 332-0012, Japan

**Keywords:** spin current, spin-polarized photocurrent, magneto-photogalvanic effect, magnetic metamaterial, opto-spintronics, symmetry engineering

## Abstract

Spin currents – flows of spin angular momentum without net charge – are central to next-generation spintronic technologies but remain difficult to generate and control efficiently. Magnetic metamaterials provide a powerful platform, as engineered structures allow symmetry design and tailored light–matter interactions. Here, we demonstrate that lateral scaling of triangular-hole Co/Pt magnetic metamaterials exerts a strong, nonlinear influence on spin-current generation via the photogalvanic and magneto-photogalvanic effects. By systematically varying the pattern size, we observe unexpected behaviors: sign reversals, and even complete suppression of photocurrents at specific wavelengths. These phenomena reveal an intimate link between optical resonance conditions and spin current generation. Our findings establish metamaterial geometry as a new degree of freedom for engineering spin currents, offering dynamic tunability of magnitude, and sign – an essential step toward tunable, optically controlled spintronic devices.

## 1 Introduction

Spintronics leverages the electron spin degree of freedom to achieve energy-efficient information processing, underpinning practical technologies such as magnetoresistive random access memory [1]. A key challenge in advancing this field is the reliable generation and control of spin currents [2], [3]. Optical approaches are particularly attractive for ultrafast, contactless spintronics, enabling polarization-based control with sub-picosecond response times [4], [5], [6].

Recently, triangular-hole Co/Pt magnetic metamaterials (MMs) with threefold rotational symmetry demonstrated polarization-dependent spin-polarized photocurrents at room temperature [7], [8]. Here, the term “spin-polarized photocurrents” refers to the nature of the generation mechanism rather than the spin properties of the carriers at the electrodes; see Section 2.2 for details. This proof-of-principle showed that symmetry dictates both the direction and magnitude of spin currents. However, a central question remains unanswered: how does the lateral scaling of nanostructures – at fixed composition and thickness – influence the magnitude, sign, and polarization dependence of spin currents? While the size dependence of engineered structures has been extensively investigated for optical phenomena such as extraordinary optical transmission (EOT) [9], [10], [11], [12], [13], [14] and optical second-harmonic generation [15], [16], [17], [18], [19], [20], its role in photo-induced spin current conversion has remained largely unexplored.

In this study, we address this gap by systematically investigating the scaling-dependent response of spin-driven photocurrents in triangular-hole Co/Pt MMs, with hole sizes ranging from 200 nm to 1,500 nm, while maintaining the metal-fill factor constant. We find that nanostructure scaling dramatically alters both the magnitude and sign of spin-polarized photocurrents, producing resonance-linked enhancements, sign reversals, and even suppression at specific wavelengths. Polarization-dependent measurements allow clear separation of photogalvanic and magneto-photogalvanic contributions, while wavelength-dependent studies suggest a possible connection to EOT-like resonances. In addition, the magneto-photogalvanic response reproduces magnetic hysteresis loops, providing a purely electrical probe of ferromagnetic switching in noncentrosymmetric systems. Our results establish metamaterial geometry as a new degree of freedom for spin-current engineering, enabling precise control over magnitude, and sign. This geometry-dependent tunability opens a versatile pathway for optically tunable spintronic devices, bridging photonics and spintronics and advancing the design of ultrafast, noncontact spin current sources.

## 2 Results and discussion

### 2.1 Photogalvanic and magneto-photogalvanic effects

A central advantage of spin-polarized photocurrents lies in their detection through entirely electrical means. To exploit this property, we employ the photogalvanic effect (PGE), which generates DC photocurrents in media lacking space inversion symmetry P . The photocurrent can be expressed as [21]

(1) $$J_{i}=\beta_{ijk}^{PGE}\left(0;\omega ,-\omega \right)E_{j}\left(\omega \right)E_{k}\left(-\omega \right),$$

where *E*_*j*_(*ω*) and *E*_*k*_(−*ω*) denote the components of the incident electric field polarized along the *j*- and *k*-directions, respectively, at frequency *ω*. The resulting zero-bias photocurrent *J*_*i*_ flows along the *i*-direction and scales quadratically with the electric-field amplitude, i.e., with the incident light intensity. The third-rank polar tensor *β*_*ijk*_, determined by the crystalline symmetry of the material, changes sign under space inversion operation.

When, in addition to broken P , time-reversal symmetry T is also broken, as in magnetic systems, the magneto-photogalvanic effect (MPGE) emerges [7], [22], [23]:

(2) $$J_{i}=\beta_{ijk}^{MPGE}\left(0;\omega ,-\omega \right)E_{j}\left(\omega \right)E_{k}\left(-\omega \right).$$

Here, $\beta_{ijk}^{MPGE}$ is a third-rank polar tensor determined by the magnetic point group, and it changes sign under both space inversion and time-reversal operations. In our system, the photocurrent is inherently spin-polarized due to the spin-imbalanced electronic structure of the magnet. This offers a direct and efficient means to switch the direction of spin-polarized photocurrents by flipping the magnetization.

### 2.2 Magnetic metamaterial with threefold rotational symmetry

**Figure 1:**
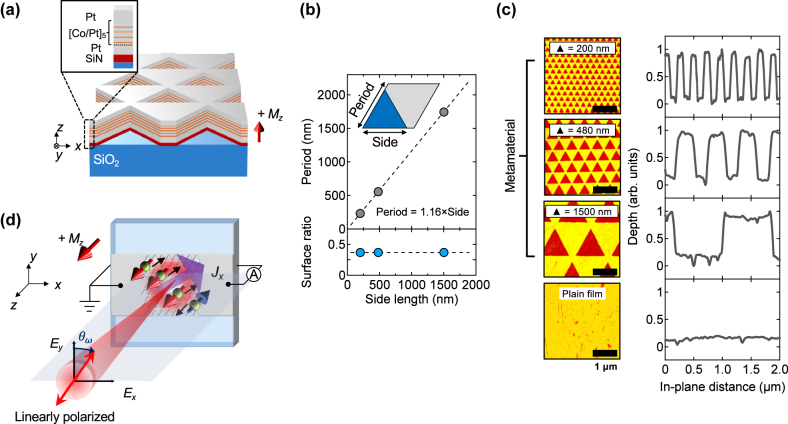
Sample design and experimental setup. (a) Schematic of a centrosymmetric Co/Pt ferromagnetic multilayer with out-of-plane magnetization *M*_*z*_, patterned into a triangular-hole lattice. The inset illustrates the multilayer structure (see Section 4 for details). (b) Definition of the MM unit cell. The lattice period scales with the side length of the triangular holes while maintaining a constant metal-fill factor across all samples. Fabricated MMs have side lengths of 200 nm, 480 nm, and 1,500 nm, corresponding to lattice periods of 233 nm, 558 nm, and 1,744 nm, respectively. (c) AFM images of the three MMs and a reference plain film (left), along with the corresponding normalized cross-sectional profiles (right), confirm the designed triangular geometry and hole structure. (d) Schematic of the experimental setup used to measure the zero-bias photocurrent *J*_*x*_, which varies with the polarization of the incident light.

To achieve simultaneous breaking of P and T , we designed magnetic metamaterials (MMs) composed of arrays of triangular holes patterned into a perpendicularly magnetized Co/Pt multilayer [7] (Figure 1(a)). The triangular geometry breaks P , which is otherwise present in Co and Pt, while the out-of-plane magnetization breaks T , thereby satisfying the symmetry conditions for both PGE and MPGE (see Section 4 for details). As a control, we also prepared an unpatterned, centrosymmetric Co/Pt multilayer sample.

Once the magnetic point group of the MMs is specified, the symmetry-allowed components of $\beta_{ijk}^{PGE}$ and $\beta_{ijk}^{MPGE}$ can be identified for each polarization of the incident field (Table 1). In our design, each effect is governed by a single independent component – one for PGE and one for MPGE – leading to zero-bias in-plane photocurrents [24]. For clarity, we denote these components simply as *β* ^PGE^ and *β* ^MPGE^.

**Table 1:** Symmetry-allowed tensor components for PGE and MPGE responsible for generating zero-bias in-plane photocurrents in MMs with threefold rotational symmetry and perpendicular magnetization.

*β* ^PGE^ ≡ $\beta_{xxy}^{PGE}=\beta_{xyx}^{PGE}=\beta_{yxx}^{PGE}=-\beta_{yyy}^{PGE}$
*β* ^MPGE^ ≡ $-\beta_{xxx}^{MPGE}=\beta_{xyy}^{MPGE}=\beta_{yxy}^{MPGE}=\beta_{yyx}^{MPGE}$

In metamaterials, the characteristic size of the nanostructure is typically smaller than the wavelength of light. To systematically examine this regime, we fabricated MMs with triangular patterns with side lengths 200, 480, and 1,500 nm (Figure 1(b)). Each triangle resides within a unit cell whose period scales with the side length, thereby keeping the metal-fill factor constant across all samples. This design ensures equivalent illumination conditions. Atomic force microscope (AFM) images confirm the intended geometry of the holes (Figure 1(c)).

Figure 1(d) illustrates the experimental setup. Under linearly polarized excitation, a photocurrent along the *x* direction is detected. Due to the exchange-split band structure of Co/Pt multilayer, conduction electrons are intrinsically spin-imbalanced, exhibiting a spin polarization of about 50 % near the Fermi level [25]. As a result, spin-polarized photocurrents are generated in the patterned area upon illumination. However, in Co and Pt, the spin diffusion length is several orders of magnitude shorter (a few nanometers) than the electrode separation (hundreds of micrometers), resulting in only charge currents being detectable at the contacts. Thus, in our discussion, the term “spin-polarized photocurrents” refers to the nature of the generation mechanism rather than the spin properties of the carriers at the electrodes.

Finally, by expressing the incident electric field as **E** = *E*_0_(sin*θ*_*ω*_, cos*θ*_*ω*_, 0), the photocurrent is written as

(3) $$J_{x}=-E_{0}^{2}\left[\beta^{PGE}\;\sin\left(2\theta_{\omega }\right)+\beta^{MPGE}\;\cos\left(2\theta_{\omega }\right)\right],$$

clearly demonstrating the separation of PGE and MPGE through their distinct polarization dependencies.

### 2.3 Magnetic characterization

**Figure 2:**
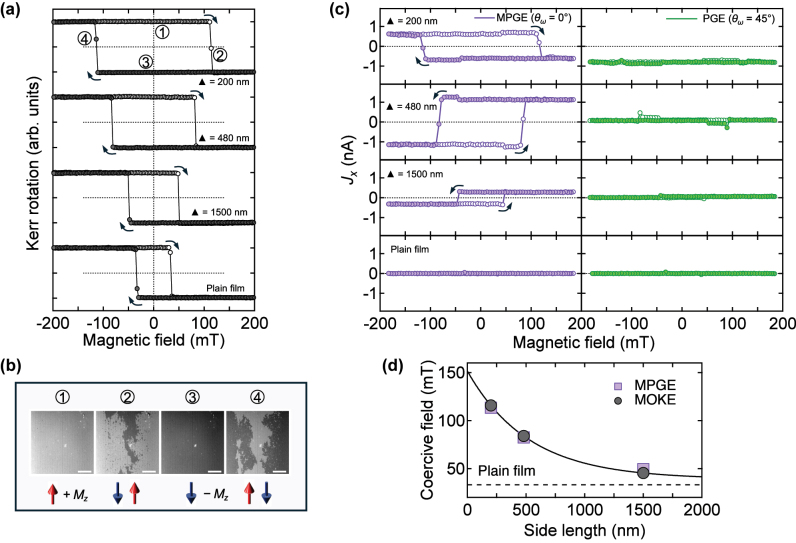
Magnetic characterization and photocurrent response of Co/Pt magnetic metamaterials. (a) Polar MOKE measurements show that the coercive field strongly depends on the nanostructure size, being largest for the 200 nm MM and smallest for the 1,500 nm MM, while all samples retain ferromagnetic behavior. (b) Representative magnetic domain images of the 200 nm MM (scale bar: 50 μm). Opposite domains appear only near the coercive field, whereas at zero field, the sample is in a single-domain state – the condition under which spin-polarized photocurrents were generated. Bright and dark contrasts correspond to up and down magnetizations, respectively. The bright dot at the center is a nonmagnetic defect on the surface. (c) Zero-bias photocurrent *J*_*x*_ as a function of external magnetic field for MPGE (left) and PGE (right), measured at normal incidence with linearly polarized 880 nm light. MPGE shows a strong field dependence, while the PGE contribution is essentially field-independent. No photocurrent is observed in the centrosymmetric plain film. (d) Comparison of coercive fields extracted from MOKE (closed circles) and MPGE (closed squares) measurements. Coercivity in MMs approaches that of the plain film (dotted line) as hole size increases; the line is a guide to the eye.

Nanoscale patterning of the Co/Pt multilayer suppresses domain nucleation and expansion, thereby modifying the plain film’s magnetic properties [26], [27]. To investigate this effect, we performed magneto-optical Kerr effect (MOKE) measurements in polar geometry (Figure 2(a)).

The MOKE results reveal a systematic increase in coercivity as pattern size decreases. Compared to the unpatterned film, the coercive field of the 200 nm MM is nearly four times higher, while maintaining clear ferromagnetic behavior. Figure 2(b) shows snapshots of the saturated state and the formation of magnetic domains in the 200 nm MM, confirming complete saturation in both magnetization directions at zero field. Similar saturated and transitional states were observed across all samples. The observed coercivity enhancement in triangular holes (antidots) arrays is consistent with prior studies attributing magnetic hardening to edge-induced domain pinning, shape anisotropy, and configurational effects arising from antidot geometry and lattice symmetry, as established through micromagnetic simulations and experimental observations [28], [29].

Because of its magnetic origin, MPGE can in principle serve as an electrical probe of magnetic hysteresis. According to Eq. (3), the MPGE contribution is isolated at *θ*_*ω*_ = 0° (left panels in Figure 2(c)), while the PGE contribution is detected at *θ*_*ω*_ = 45° (right panels in Figure 2(c)). Under 880 nm irradiation, the hysteresis loop of the 200 nm MM inverts relative to that of larger-period MMs, suggesting that the sign of the spin-polarized photocurrent depends on nanostructure size. Nevertheless, ferromagnetic hysteresis is clearly observed in all samples (see Appendix for details on the two-step features near coercive fields). By contrast, the centrosymmetric film shows no response, consistent with the absence of MPGE or PGE.

Notably, coercive fields extracted from MOKE and MPGE measurements are in excellent agreement (Figure 2(d)). As the triangular-hole size decreases, domain expansion is progressively hindered, resulting in higher switching fields. These findings establish MPGE as a reliable electrical probe of ferromagnetic hysteresis, offering an alternative optical approach to investigate noncentrosymmetric magnets.

### 2.4 Polarization dependence of MPGE and PGE

**Figure 3:**
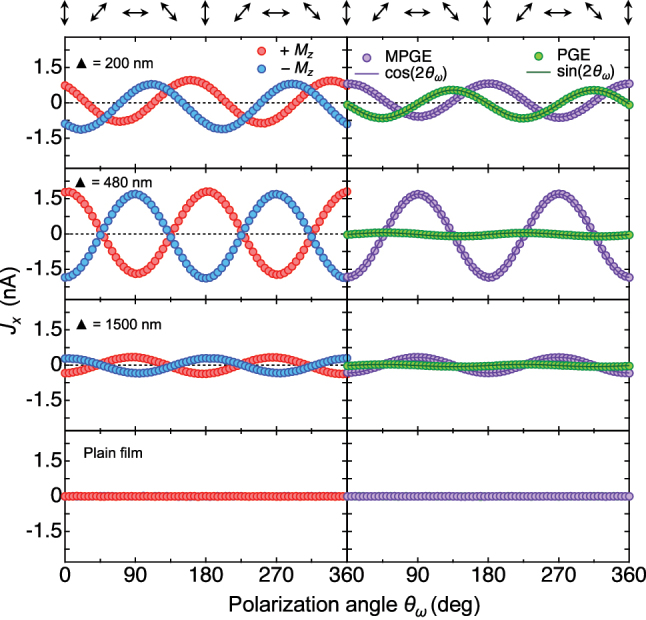
Polarization dependence of MPGE and PGE at 880 nm. The zero-bias photocurrent *J*_*x*_ shows a clear dependence on the polarization angle *θ*_*ω*_ of the incident light, consistent with symmetry predictions. In all MMs except the 200 nm one, the photocurrents at opposite magnetization directions are nearly equal in magnitude but opposite in sign. Subtracting the signals from opposite magnetizations isolates the MPGE contribution, whereas their sum corresponds to the magnetization-independent PGE. Under 880 nm irradiation, the MPGE amplitude is comparable to or larger than that of the PGE in all MMs. As expected, no photocurrent is detected in the centrosymmetric plain film.

Magnetic characterization of the MMs already suggested the coexistence of MPGE and PGE at certain polarization angles. To further verify the relation in Eq. (3), we systematically examined the full polarization-angle dependence of the zero-bias photocurrent at 880 nm in all samples (left panels in Figure 3). Prior to measurement, we saturated each sample in both magnetization directions and then measured photocurrents at zero external field.

At arbitrary polarization angles, MPGE and PGE signals overlap: summing currents under opposite magnetizations isolates the magnetization-independent PGE contribution, while their difference isolates the pure MPGE signal (right panels in Figure 3). In the 200 nm MM, photocurrent traces at opposite magnetizations are not perfectly symmetric, indicating a significant PGE contribution. By contrast, the 480 nm and 1,500 nm MMs show nearly symmetric responses, revealing a negligible PGE. The extracted MPGE and PGE components agree well with Eq. (3) (dark violet and green lines), confirming the validity of the model. As expected, the centrosymmetric plain film shows no photocurrent at any polarization angle, since the symmetry requirements for both MPGE and PGE are not fulfilled.

### 2.5 Wavelength dependence of MPGE and PGE

**Figure 4:**
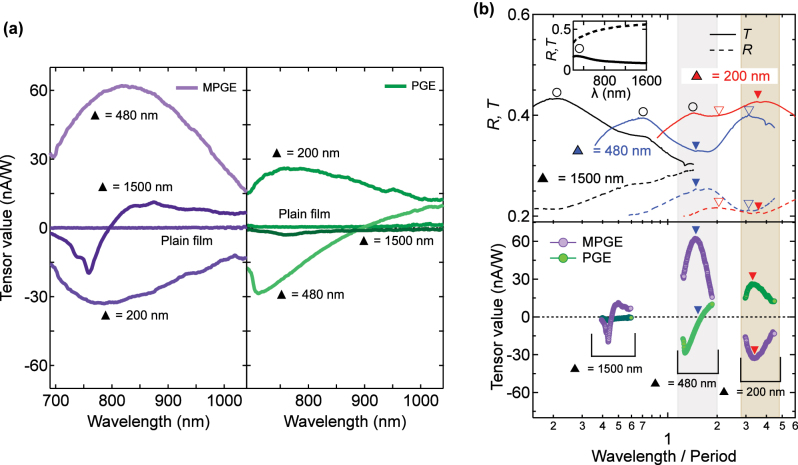
Wavelength dependence of MPGE and PGE tensors and their relation to optical spectra. (a) Dependence of *β* ^MPGE^ (left) and *β* ^PGE^ (right) on excitation wavelength for MMs with triangular side lengths of 200, 480, and 1,500 nm. In the 1,500 nm MM, MPGE vanishes near 800 nm, whereas PGE vanishes near 900 nm in the 480 nm MM. In contrast, the 200 nm MM retains both effects with opposite sign relative to the larger MMs. (b) Linear transmission (solid lines) and reflection (dotted lines) spectra normalized by lattice period (top). Corresponding normalized MPGE and PGE spectra (bottom) show that extrema and zero-crossings coincide with EOT-like features (closed triangles). Shaded areas serve as visual guides. Transmission peaks in the shorter-wavelength region (open circles) are present even in the plain film (inset), indicating that they are unrelated to the nanopatterned structure. The overlap between resonance features and photocurrent modulation highlights the key role of resonant optical coupling in tuning MPGE and PGE.

Results at 880 nm already highlighted a strong dependence of the photocurrent on both nanostructure size and excitation wavelength. To comprehensively investigate this behavior, we measured MPGE and PGE across the 690–1,040 nm range in 2 nm increments (Figure 4(a)).

The sign and magnitude of the tensor components vary significantly among samples. For example, the MPGE of the 200 nm and 480 nm MMs remains finite but has opposite sign across the full spectral range, while the 1,500 nm MM exhibits a sign reversal around 796 nm after an initial minimum at 758 nm. This size- and wavelength-dependent inversion suggests that structural scaling can suppress MPGE even under conditions where it is symmetry-allowed. Overall, the MPGE amplitude is highest in the 480 nm MM, reaching nearly twice the level of the 200 nm MM and four times that of the 1,500 nm MM, particularly in the 750–850 nm region. The plain film yields no photocurrent across all wavelengths.

The PGE also shows complex spectral evolution. In the 480 nm MM, it develops a minimum at 710 nm, vanishes near 900 nm, and reverses sign at longer wavelengths. In the 200 nm MM, the PGE intensity is comparable to that of the 480 nm MM at shorter wavelengths but retains opposite sign relative to the larger structures. Importantly, MPGE and PGE never vanish simultaneously, and the absolute MPGE value is consistently equal to or larger than the corresponding PGE at any wavelength. These features cannot be explained solely by simple asymmetric scattering models [21], requiring further insight on the optical properties of the MMs.

### 2.6 Extraordinary optical transmission and resonance coupling

To clarify the role of optical resonances, we compared the photocurrent spectra with the linear transmission and reflection spectra normalized by the lattice period (Figure 4(b)). In the 200 nm and 480 nm MMs, transmission dips coincide with reflection peaks (closed and open triangles), characteristic of EOT reported in both plasmonic and lossy metals [30], [31]. In the 1,500 nm MM, however, EOT-like features are not clearly observed within the measured spectral range, suggesting that its resonance may be either weaker or shifted outside the measurement window. All samples, including the centrosymmetric reference film, exhibit a transmission peak at shorter wavelengths (open circles), attributable to the intrinsic property of the Co/Pt multilayer rather than nanopatterning.

The extrema and zero-crossings of the photocurrent spectra frequently align with these optical resonances. In the 480 nm MM, the EOT dip (blue closed triangles) coincides with a maximum in MPGE and a vanishing PGE, consistent with earlier predictions for subwavelength hole arrays [15]. In the 200 nm MM, by contrast, both PGE and MPGE peak at the EOT maximum (red closed triangles), underscoring the role of plasmon-enhanced near-fields. These fields are strongly localized at the triangular edges and vertices, where steep electric-field gradients form [32]. Such localized fields may enhance both PGE and MPGE, contributing to the spectral profiles observed across the three lattice periods. These observations suggest that resonant optical modes could play an important role in generating spin-polarized photocurrents in MMs.

In particular, the interplay of field enhancements and symmetry breaking represents a promising framework that could benefit from further theoretical modeling to establish a more complete understanding. Existing studies of this type have so far been limited to crystalline materials with atomic-scale unit cells, such as CrI_3_ [23], [33] and MnBi_2_Te_4_ [34], which differ by many orders of magnitude from the artificial-symmetry unit cells of our MMs. Thus, scaling-dependent MM systems represent a promising platform for both experimental and theoretical investigations.

## 3 Conclusions

We have demonstrated that spin-polarized photocurrents can be effectively tuned by scaling the hole size in perpendicularly magnetized Co/Pt MMs. Whereas plain films do not generate photocurrents, the triangular-hole lattices consistently produce spin-polarized photocurrents. Moreover, MPGE proved to be a powerful electrical probe of magnetism in noncentrosymmetric magnets. By systematically varying the excitation wavelength, we found that both the magnitude and the sign of photocurrents exhibit nontrivial dependence on the nanostructure size (lattice period). Importantly, MPGE and PGE never vanish simultaneously, and the MPGE amplitude consistently exceeds that of the PGE. These results highlight that nanoscale scaling governs not only the efficiency but also the polarity of photocurrent generation. Furthermore, the observed coupling between resonant optical modes and photocurrent generation points to a possible relationship between EOT and plasmonic enhancement in tailoring spin-polarized photocurrents in MMs.

Taken together, our findings establish nanoscale hole patterning as a versatile route to realizing spin-current sources that are noncontact, polarization-tunable, and wavelength-sensitive. Future efforts should focus on elucidating the underlying dynamics through advanced electromagnetic simulations and near-field optical microscopy, as well as extending PGE and MPGE studies across a broader class of materials. Such approaches hold promise for optimizing metamaterial designs and unlocking new functionalities in ultrafast spintronics and opto-spintronic devices.

## 4 Methods

**Fabrication of magnetic metamaterials:** To satisfy the symmetry conditions required for observing both PGE and MPGE, we designed MMs that simultaneously break P and T by introducing triangular hole arrays into a Co/Pt ferromagnetic multilayer. Each MM consists of a periodic triangular-hole lattice with threefold rotational symmetry, patterned on a double-polished SiO_2_ substrate. The multilayer stack was deposited by RF magnetron sputtering and comprises 5 nm SiN, 2 nm Pt, a [Pt (0.9 nm)/Co (0.5 nm)] × 5 multilayer, and a 2 nm Pt capping layer. Triangular holes were defined across a 250 × 250 μm^2^ area using electron-beam lithography followed by Ar-ion etching.

We fabricated four samples: three MMs with triangular side lengths of 200 nm, 480 nm, and 1,500 nm (corresponding to lattice periods of 233 nm, 558 nm, and 1,744 nm, respectively), and one unpatterned plain film used as a control. AFM images confirmed the intended geometry and high structural quality of all fabricated samples. The triangular unit cell belongs to the 3*m* point group with threefold symmetry. At room temperature, the Co/Pt multilayer exhibits perpendicular magnetization, preserving threefold rotational symmetry while breaking T (magnetic point group 3*m*′). As a result, both P and T are broken, fulfilling the requirements for the emergence of PGE and MPGE.

**Magneto-optical Kerr effect measurements:** The response of each MM to an external magnetic field was characterized using a MOKE microscope in polar geometry. The nanoscale patterning of the MMs modifies domain nucleation and growth, resulting in an enhanced coercive field that increases as the triangular hole size decreases.

**Photocurrent measurements:** Using the surrounding unstructured Co/Pt film as electrodes, we measured short-circuit photocurrents in both the MMs and the plain film. All measurements were performed at room temperature under ambient conditions and at normal incidence. Excitation was provided by a tunable Ti:sapphire laser (Spectra-Physics, Mai Tai HP) operating from 690 to 1,040 nm, delivering 100 fs pulses at 80 MHz. A rotatable half-wave plate controlled the polarization angle *θ*_*ω*_ of the linearly polarized incident light (Figure 1(d)), and the beam was focused to a ∼250 μm spot to illuminate the nanostructured region. The beam intensity was modulated at *f* ∼ 1 kHz with a mechanical chopper, and zero-bias photocurrents along the *x* direction were detected using a two-phase lock-in amplifier (SR865, Stanford Research Systems).

**Linear optical measurements:** The linear optical transmission (*T*) and reflection (*R*) spectra of the reference plain Co/Pt multilayer film and the MMs were measured in the 250–2,500 nm range using a commercial spectrometer (JASCO MSV-5700).

## Appendix: Contribution of the anomalous Nernst effect to photocurrent

In addition to MPGE and PGE, the two-step change observed in the hysteresis loop near the coercive fields – visible only in electrical measurements – can be attributed to heat-related effects at the boundary between patterned and unpatterned regions (see Figure 1(d)). We attribute this feature to the anomalous Nernst effect (ANE) [35], [36], expressed as

(4)
$$E_{N}\propto M\times \left(-\nabla T\right),$$

where **E**_N_ denotes the Nernst electric field, **M** is the magnetization, and −∇*T* represents the temperature gradient across the sample. Assuming a temperature gradient along the *y* direction at the patterned–unpatterned boundary, an **E**_**N**_ arises along the *x* direction, thereby adding to the MPGE and PGE photocurrents. This contribution becomes evident in the field interval between the coercive field of the plain film (about 33 mT) and that of each MM, where the two regions exhibit opposite magnetization along the *z* direction. Within this interval, the ANE contribution points in the same direction as MPGE and PGE, contributing to the total current measurement. Because the ANE scales with magnetization and unconstrained by symmetry, it manifests as a minor yet visible contributions to the overall photocurrent.
